# Supplementary material for: Different Disease Levels Reveal Kiwifruit Brown Spot Impacts on Fruit Yield and Quality
Source: J Fungi (Basel). 2025 Aug 15;11(8):593. doi: 10.3390/jof11080593 (PMC12387563; doi:10.3390/jof11080593)
Supplement: Supplementary file 1 [file jof-11-00593-s001.zip › Figure S1.pdf]

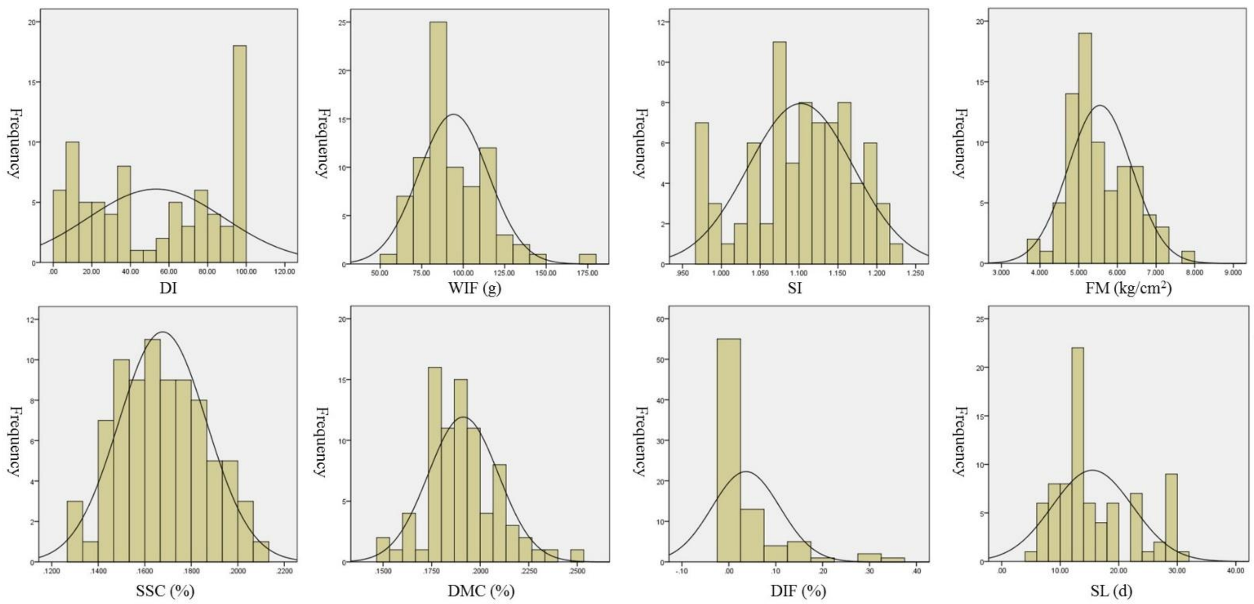

Figure. S1 Range and frequency of fruit quality indices in different disease level.
DI: disease index, WIF: weight of individual fruit, SI: shape index, FM: firmness, SSC: soluble solids content, DMC: dry matter content, DIF: disease incidence of fruit rot, SL: shelf life
