# Supplementary material for: Different Disease Levels Reveal Kiwifruit Brown Spot Impacts on Fruit Yield and Quality
Source: J Fungi (Basel). 2025 Aug 15;11(8):593. doi: 10.3390/jof11080593 (PMC12387563; doi:10.3390/jof11080593)
Supplement: Supplementary file 1 [file jof-11-00593-s001.zip › Table S1.pdf]

Table S1. The corresponding equation of each plot based on the firmness and soluble solid content.

Plot	Equation	R^2	F Value
AZ01	y=7.05+13.37/[1+exp((x-2.06)/0.97)]	0.952	1041.69
AZ02	y=8.74+25.91/[1+exp((x+0.25)/1.65)]	0.969	1926.35
AZ03	y=6.13+10.27/[1+exp((x-4.27)/0.032)]	0.932	534.03
AZ04	y=7.60+14.49/[1+exp((x-1.71)/0.98)]	0.962	1229.51
AZ05	y=7.08+11.87/[1+exp((x-3.19)/0.18)]	0.986	2137.74
AZ06	y=6.96+11.99/[1+exp((x-2.92)/0.54)]	0.988	3141.84
AZ07	y=6.22+10.95/[1+exp((x-3.98)/0.12)]	0.989	2609.80
AZ08	y=6.44+12.45/[1+exp((x-2.33)/1.08)]	0.953	938.81
AZ09	y=5.52+13.82/[1+exp((x-3.24)/1.06)]	0.973	1499.09
AZ10	y=7.63+18.31/[1+exp((x-0.95)/1.15)]	0.984	1945.52
AZ11	y=-1.98+19.53/[1+exp((x-5.80)/1.91)]	0.886	523.36
AZ12	y=6.80+9.98/[1+exp((x-4.02)/1.02)]	0.878	744.99
AZ13	y=3.24+11.27/[1+exp((x-5.91)/1.26)]	0.883	590.78
AZ14	y=8.72+7.19/[1+exp((x-4.29)/0.19)]	0.886	817.48
AZ15	y=-9.56+386.29/[1+exp((x+29.91)/11.78)]	0.934	605.44
AZ16	y=6.96+9.07/[1+exp((x-3.74)/0.81)]	0.972	1684.83
AZ17	y=6.30+9.31/[1+exp((x-1.66)/0.083)]	0.986	1956.35
AZ18	y=8.15+7.82/[1+exp((x-2.37)/0.53)]	0.899	276.79
AZ19	y=3.63+12.49/[1+exp((x-4.87)/2.08)]	0.848	511.23
AZ20	y=8.46+7.65/[1+exp((x-2.80)/1.05)]	0.945	1233.19
AZ21	y=9.30+6371.60/[1+exp((x+7.43)/1.19)]	0.943	540.86
AZ22	y=5.48+8.67/[1+exp((x-5.04)/0.60)]	0.962	1104.29
AZ23	y=3.45+13.46/[1+exp((x-3.93)/2.29)]	0.971	1102.00
AZ24	y=7.38+5.43/[1+exp((x-5.10)/0.30)]	0.832	332.73
AZ25	y=7.49+11.94/[1+exp((x-4.15)/0.22)]	0.986	2753.17
AZ26	y=11.99+18.68[1+exp((x+0.11)/2.07)]	0.891	1413.64
AZ27	y=9.93+8.61[1+exp((x-4.31)/0.099)]	0.922	1010.53
AZ28	y=-574.03+788.96/[1+exp((x-120.18)/108.67)]	0.879	257.19
AZ29	y=10.22+8.02[1+exp((x-4.64)/0.27)]	0.823	424.32
AZ30	y=4.01+768.98[1+exp((x+31.77)/8.16)]	0.879	265.31
DJY01	y=3.63+14.34/[1+exp((x-4.32)/1.45)]	0.955	1333.54
DJY02	y=5.66+11.28/[1+exp((x-4.53)/0.29)]	0.983	2301.02
DJY03	y=2.88+18.39/[1+exp((x-3.75)/2.40)]	0.958	1649.17
DJY04	y=9.39+5.63/[1+exp((x-4.14)/0.096)]	0.815	725.75
DJY05	y=10.58+4.80/[1+exp((x-3.76)/0.12)]	0.867	2035.80
DJY06	y=1.46+346.46/[1+exp((x+37.18)/11.91)]	0.877	1465.29
DJY07	y=2.72+18.32/[1+exp((x-4.51)/2.36)]	0.936	1054.32
DJY08	y=-288.72+370.17/[1+exp((x-58.25)/36.63)]	0.910	918.94
DJY09	y=10.66+9.21/[1+exp((x-1.37)/0.44)]	0.962	1628.71

DJY10	y=9.75+5.92/[1+exp((x-3.63)/0.24)]	0.886	964.56
DJY11	y=8.78+2065.82/[1+exp((x+10.02)/1.87)]	0.938	1391.87
DJY12	y=-1.22+388.58/[1+exp((x+29.13)/9.78)]	0.924	1151.69
DJY13	y=9.68+5.15/[1+exp((x-3.61)/0.10)]	0.868	1433.54
DJY14	y=9.57+5.89/[1+exp((x-3.79)/0.93)]	0.901	977.25
DJY15	y=7.12+63.57/[1+exp((x+10.33)/5.66)]	0.897	1375.48
DJY16	y=-1.61+28.11/[1+exp((x-4.63)/2.73)]	0.939	945.68
DJY17	y=9.76+10.10/[1+exp((x-3.54)/0.026)]	0.945	938.12
DJY18	y=7.12+13.90/[1+exp((x-3.61)/0.90)]	0.945	990.36
DJY19	y=7.37+12.86/[1+exp((x-5.09)/0.038)]	0.992	4836.57
DJY20	y=7.90+13.30/[1+exp((x-3.88)/0.16)]	0.973	1574.91
DJY21	y=8.74+11.30/[1+exp((x-3.65)/1.01)]	0.942	1035.60
DJY22*	y=-1.4056x+20.362	0.934	
DJY23	y=5.33+16.52/[1+exp((x-4.13)/1.76)]	0.915	1646.46
DJY24	y=10.60+7.52/[1+exp((x-2.05)/0.042)]	0.961	1694.31
DJY25	y=9.14+9.79/[1+exp((x-2.84)/0.62)]	0.913	1169.35
LS01	y=3.94+13.92/[1+exp((x-4.97)/0.71)]	0.979	1932.56
LS02	y=5.97+11.24/[1+exp((x-4.04)/0.56)]	0.983	1989.43
LS03	y=5.48+11.95/[1+exp((x-3.27)/0.071)]	0.972	968.20
LS04	y=7.32+10.16/[1+exp((x-3.01)/0.077)]	0.978	1200.35
LS05	y=5.18+11.09/[1+exp((x-5.82)/0.20)]	0.979	2368.53
LS06	y=4.48+11.59/[1+exp((x-4.66)/0.70)]	0.987	2386.83
LS07	y=-303.34+819.46/[1+exp((x+56.54)/128.69)]	0.971	1281.05
LS08	y=12.27+4.98/[1+exp((x-0.61)/0.23)]	0.876	1376.84
LS09	y=-383.38+404.98/[1+exp((x-38.44)/9.73)]	0.917	1603.64
LS10	y=-15005.66+15022.53/[1+exp((x-17.15)/1.46)]	0.832	1042.22
LS11	y=-21.10+36.68/[1+exp((x-7.74)/1.36)]	0.859	1112.21
LS12	y=12.50+9.14/[1+exp((x-0.69)/0.34)]	0.949	3013.96
LS13	y=-5193.76+5208.40/[1+exp((x-19.13)/1.88)]	0.884	575.55
LS14*	y=-0.7205X+15.283	0.798	
LS15	y=9.52+5.29/[1+exp((x-3.81)/0.15)]	0.912	1309.99
LS16	y=7.30+7.95/[1+exp((x-12.72)/0.85)]	0.956	1025.57
LS17	y=-1030.28+1054.14/[1+exp((x-57.15)/11.58)]	0.826	468.27
LS18	y=13.36+3.34/[1+exp((x-0.20)/0.0019)]	0.849	665.99
LS19	y=13.32+4.53/[1+exp((x-3.68)/0.36)]	0.914	1653.44
LS20	y=4.14+16.44/[1+exp((x-5.26)/2.43)]	0.872	2038.87
LS21	y=-304.56+819.47/[1+exp((x+90.99)/207.07)]	0.902	2043.94
LS22	y=9.69+9.27/[1+exp((x-4.27)/0.0086)]	0.921	1994.71
LS23	y=-23.78+226.38/[1+exp((x+34.84)/25.01)]	0.929	1278.89
LS24	y=-3697.08+3718.64/[1+exp((x-18.95)/2.41)]	0.879	1180.34
LS25	y=9.75+9.42/[1+exp((x-3.76)/1.26)]	0.810	421.79
LS26	y=13.29+7.63/[1+exp((x-1.53)/0.84)]	0.820	1035.19

Note: * Boltzmann model did not fit converge, so replaced by linear model.
